# In Situ Inorganic Salt-Enabled Laser-Induced Graphene for High-Performance Flexible Capacitive Humidity Sensing

**DOI:** 10.3390/nano16160996

**Published:** 2026-08-13

**Authors:** Jitong Ren, Zihan Li, Lei Gu, Weilu Chen, Xinyi Zhou, Yanyan Guo, Jiang Zhao

**Affiliations:** College of Integrated Circuit Science and Engineering, Nanjing University of Posts and Telecommunications, 9 Wenyuan Road, Nanjing 210023, China

**Keywords:** humidity sensor, capacitive, lithium chloride, potassium acetate, laser-induced graphene

## Abstract

Flexible capacitive humidity sensors are pivotal for next-generation wearable electronics and Internet of Things (IoT) applications. However, conventional devices suffer from severe salt leaching and delamination of hygroscopic sensing materials, alongside poor interfacial adhesion and mechanical fragility of metallic electrodes. Herein, an innovative in situ strategy is reported for constructing LiCl-CH_3_COOK/laser-induced graphene (LIG) composite flexible electrodes via single-step laser direct writing. This approach simultaneously patterns three-dimensional (3D) porous LIG interdigitated networks on polyimide substrates and drives deep infiltration of the LiCl-CH_3_COOK hygroscopic phase within the graphene pores. The 3D interconnected LIG skeleton not only provides abundant physical anchoring sites and rapid water vapor transport channels but also effectively suppresses the physical loss and leaching of the deliquesced salts through micro-nanoscale spatial confinement, yielding remarkable interfacial stability and cycling lifetime. Benefiting from the synergistic deliquescence of the composite salts, the sensor delivers an exceptional sensitivity of 65,570% (Δ*C*/*C*_0_), moderate response/recovery times of 75/90 s, and ultralow hysteresis of 0.981%. Furthermore, the streamlined laser-scribing route replaces conventional costly microfabrication sequences, enabling low-cost, high-precision customization. Demonstrations in human respiration monitoring and smart agriculture validate the sensor’s superior reliability and practical applicability, establishing a novel pathway for miniaturized, highly integrated, and robust flexible humidity detection systems.

## 1. Introduction

Real-time and comfortable monitoring of microenvironmental humidity is a critical imperative for the expanding applications in personalized medicine, intelligent wearables, and contactless sensing [1,2,3]. Conventional rigid humidity sensors exhibit fundamental limitations in conforming to complex curvilinear surfaces and the human body. Flexible humidity sensors, distinguished by their superior mechanical extensibility and high sensitivity, have emerged as essential components in next-generation Internet of Things (IoT) and health monitoring systems [4,5,6].

Among the available humidity sensing mechanisms, capacitive humidity sensors demonstrate distinct advantages including rapid response characteristics, low power consumption, high linearity, minimal temperature drift, and excellent compatibility with mainstream microelectronic manufacturing processes [7]. This sensing modality successfully addresses the inherent deficiencies of alternative approaches, specifically the substantial temperature drift observed in resistive sensors, the frequency interference susceptibility of impedance-based sensors, and the prohibitively large form factors of optical devices. Consequently, capacitive sensing has established itself as the predominant approach in flexible humidity detection applications [8,9,10].

The humidity-sensitive material constitutes the fundamental determinant of detection limits and reliability in capacitive sensing devices. Among existing materials, ceramic phases are inherently incompatible with flexible applications owing to their requirement for high-temperature sintering and their lack of mechanical compliance [11]. Polymer matrices, although advantageous for film formation, are susceptible to severe swelling and hysteresis under high-humidity conditions [12]. Two-dimensional (2D) nanosheets, exemplified by graphene oxide and MXene, possess abundant active sites and large specific surface areas, yet inevitably undergo interlayer restacking and irreversible surface chemical reconstruction during prolonged hygrothermal cycling [13,14]. These intrinsic material deficiencies collectively result in sluggish device response, pronounced baseline drift, and reduced operational lifetime. Inorganic salts represent another important class of humidity-sensitive materials owing to their strong hygroscopicity and well-defined ionic conduction mechanisms. However, single-component salt systems often suffer from limited operational range and suboptimal sensitivity, as a single salt cannot simultaneously deliver high hygroscopicity across the entire humidity spectrum and long-term stability. To address these longstanding challenges, a composite inorganic salt system of lithium chloride and potassium acetate (LiCl-CH_3_COOK) emerges as a superior alternative, distinguished by its exceptional physicochemical characteristics. This composite exhibits not only pronounced hygroscopic activity and a well-defined hydrated ionic conduction network but also undergoes rapid deliquescence upon humidity fluctuation through reversible adsorption and desorption of water molecules, thereby liberating a substantial population of mobile ions. This process drives a nonlinear transition in both the macroscopic dielectric constant and electrical conductivity of the material [15,16]. The composite system demonstrates exceptionally high intrinsic sensitivity and signal fidelity across the entire humidity range, positioning it as an ideal candidate for constructing stable sensing layers.

The full realization of humidity-sensitive material performance necessitates a highly compatible flexible electrode. Conventional fabrication approaches predominantly rely on physical vapor deposition (PVD) methods, exemplified by electron beam evaporation [17] and magnetron sputtering [18], combined with complex photolithography to construct noble metal interdigitated electrodes on polymer substrates. This technological route suffers from high costs, cumbersome processing steps, and critically weak interfacial adhesion between the metal layer and the flexible substrate. Such weak bonding readily induces microcrack formation or complete delamination under dynamic stretching or bending. Moreover, this rigid fabrication paradigm fails to meet the demand for cost-effective, large-area production of microscale electrode arrays on flexible curved surfaces. The emergence of laser-induced graphene (LIG) provides an ideal solution for integrated electrode-substrate design in flexible humidity sensors [19,20,21,22]. This technique employs laser scribing to in situ generate three-dimensional (3D) porous graphene networks on the surface of polymers such as polyimide (PI) [19]. This process achieves high-precision patterning of the conductive network in a single step, obviating the need for complex procedures like photolithography, transfer, or chemical reduction. The method substantially simplifies the fabrication workflow of traditional graphene devices and markedly reduces manufacturing costs. LIG itself possesses a continuous and interconnected 3D porous architecture, combining excellent intrinsic electrical conductivity with robust mechanical flexibility [23]. Its exceptionally high specific surface area furnishes abundant anchoring sites for the humidity-sensitive material, while its through-connected pore channels create rapid transport pathways for water vapor adsorption and diffusion [24]. Recently, LIG has been successfully integrated into flexible humidity sensors through various strategies. For instance, Ren et al. demonstrated a LIG-anchored GO-PEDOT:PSS-MXene ternary composite humidity sensor, wherein the sensing gel was drop-cast onto pre-fabricated LIG interdigitated electrodes, achieving high sensitivity through a moisture-driven swelling mechanism [9]. He et al. prepared a flexible capacitive humidity sensor using plasma-modified graphene oxide (P-GO) as the sensing layer and LIG interdigitated electrodes transferred onto a PDMS substrate. Ar/EtOH/O_2_ plasma treatment enriched surface oxygen-containing functional groups and roughened the GO film, achieving nearly 10-fold higher sensitivity than pristine GO devices [25]. Huang et al. developed a capacitive humidity sensor with enhanced sensitivity based on a GO/lignosulfonate (GO/LS) composite, wherein LIG interdigitated electrodes were fabricated on NOMEX polyimide composite paper via laser direct writing technology, and then the GO/LS sensing film was drop-coated onto the pre-prepared electrodes [26]. Li et al. reported a paper-based humidity sensor employing screen-printed LIG conductive ink and potassium acetate-infused paper as the hygroscopic layer [16]. While these studies have established the viability of LIG as an electrode platform for humidity sensing, they both follow a sequential, post-deposition fabrication paradigm in which the sensing material is applied onto the LIG surface after electrode formation. This approach inherently limits the degree of integration between the sensing material and the LIG scaffold, as the material resides primarily on the surface rather than being deeply embedded within the 3D porous network. Consequently, LIG functions as an ideal electrode and loading scaffold for flexible humidity-sensing devices.

Despite considerable advancements in flexible humidity sensors, devices based on inorganic salts remain constrained by fundamental physical and fabrication challenges. Conventional device architectures rely on a sequential process where electrodes are fabricated first, followed by the deposition or immersion of the humidity-sensitive material [27]. This approach results in only weak physical adsorption between the ionic salt and the flexible electrode, with an absence of robust chemical bonding or effective spatial confinement. Consequently, the LiCl-CH_3_COOK composite is highly susceptible to leaching through deliquescence and to delamination during dynamic flexure or under cyclic high-humidity exposure. These phenomena induce significant response drift and can culminate in complete device failure. Furthermore, traditional metal interdigitated electrodes rely heavily on photolithography and vacuum deposition, processes that incur high fabrication costs. The resulting thin metallic films exhibit poor adhesion to flexible polymer substrates and are prone to cracking under mechanical deformation. These limitations hinder the achievement of low-cost, high-consistency, micrometer-scale mass manufacturing required for advanced flexible sensing applications. Moreover, device structures produced via this sequential methodology struggle to simultaneously satisfy the criteria of superior mechanical flexibility, bending stability, and low-cost manufacturability. This incompatibility prevents their integration into modern wearable electronics, which impose stringent demands for miniaturized and highly integrated sensing units.

To address these bottlenecks, building upon prior LIG-based humidity sensor platforms [9,16], the present work introduces a fundamentally different fabrication strategy. Specifically, this study proposes a novel in situ precursor-mixing route for constructing LiCl-CH_3_COOK/LIG composite flexible electrodes, successfully developing a new generation of high-performance capacitive humidity sensors. This strategy focuses on integrated device construction and multidimensional synergy, utilizing single-step laser direct writing to precisely pattern 3D porous LIG interdigitated networks on PI substrates while simultaneously inducing deep infiltration and spatial confinement of the LiCl-CH_3_COOK humidity-sensitive phase within the graphene pores. This composite paradigm generates remarkable synergistic benefits: the 3D interconnected LIG skeleton not only provides abundant physical anchoring sites for the composite inorganic salts but also effectively eliminates the physical leaching and loss of the deliquesced LiCl-CH_3_COOK during long-term operation through micro-nanoscale spatial confinement, thereby significantly enhancing interfacial bonding strength and substantially extending cycling lifetime. Furthermore, the excellent charge transport capability of the LIG network substantially reduces interfacial contact impedance, ensuring low-loss output of sensing signals. This strategy employs a streamlined processing route that effectively replaces conventional lengthy microfabrication sequences, enabling low-cost, high-volume customization of high-precision electrodes and high-performance humidity-sensitive units, thus establishing a novel pathway for miniaturization, integration, and highly reliable design of flexible humidity detection systems.

## 2. Experiment

### 2.1. Materials

The full complement of inorganic salts required for this investigation (namely, CH_3_COOK, MgCl_2_, KCl, K_2_CO_3_, K_2_SO_4_, NaBr, CuCl_2_, NaCl, and LiCl) was supplied by Aladdin Biochemical Technology Co., Ltd., Shanghai, China and Sinopharm Chemical Reagent Co., Ltd., Shanghai, China. A commercial thermoplastic polyamic acid (PAA) solution, characterized by its brown, transparent appearance, was obtained from Furunte Plastic New Material Co., Ltd., Changzhou, China. This solution possessed a solids content of 15–20% and a density of 1.0–1.1 g cm^−3^. For the substrate, a 75 μm thick Kapton-type PI film was provided by Beilong Electronic Materials Co., Ltd., Guangzhou, China. Throughout all procedures, highly purified water was utilized. All chemical reagents, being of analytical grade, were used in their as-received condition, with no subsequent purification steps being performed.

### 2.2. Preparation of the Flexible LiCl-CH_3_COOK/LIG Humidity Sensor

**Figure 1:**
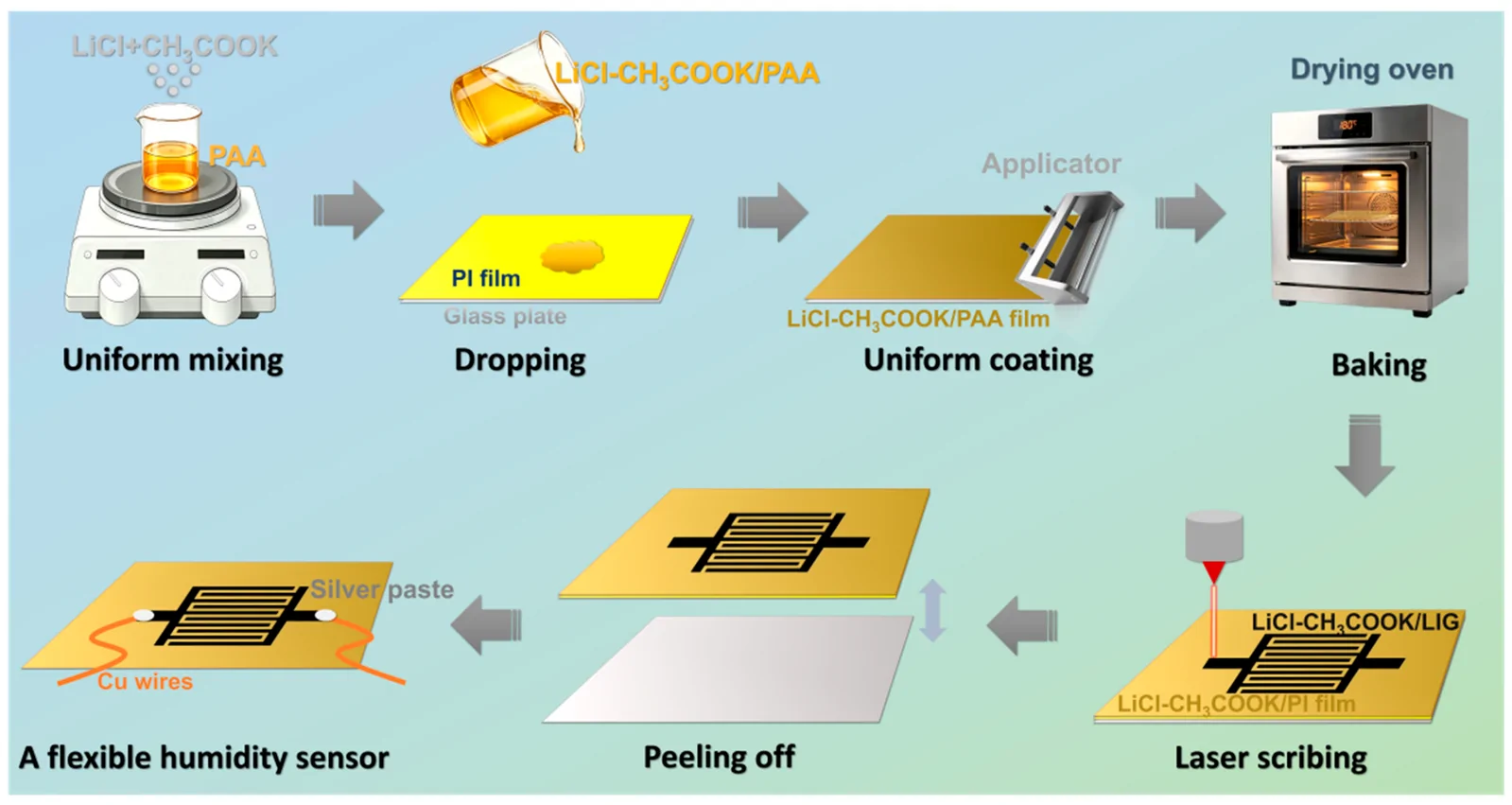
A schematic representation of the manufacturing protocol for the flexible LiCl-CH_3_COOK/LIG humidity sensor.

Figure 1 presents the fabrication schematic of the flexible LiCl-CH_3_COOK/LIG humidity sensor. The detailed procedure was as follows: dried LiCl powder (0.1 g) and varying amounts of dried CH_3_COOK powder (0.01, 0.03, 0.05, or 0.07 g) were sequentially added into a beaker containing PAA solution (10 g). The mixture was stirred continuously for 3 h at room temperature to ensure complete dispersion of both inorganic salts and uniform mixing with the PAA solution, forming a viscous LiCl-CH_3_COOK/PAA blend. The resulting solution was subsequently placed in a vacuum degassing chamber for 40 min to eliminate air bubbles generated during stirring. A 75 μm thick PI film was selected and smoothly affixed onto a glass plate, and secured with tape around its edges to prevent thermal shrinkage and surface wrinkling during the subsequent laser ablation. The LiCl-CH_3_COOK/PAA blend was then dispensed onto the PI film and blade-coated at a constant speed along one edge to form a gel layer with a thickness of 50 μm. The coated assembly was transferred to a vacuum drying oven and heated at 180 °C for 1 h, whereupon imidization occurred to yield a LiCl-CH_3_COOK/PI composite film. A CO_2_ laser scribing system, operating at 90 W power and 110 mm s^−1^ scanning speed, was employed to scribe a pre-designed interdigitated electrode pattern onto the LiCl-CH_3_COOK/PI composite film surface. The specific parameters of the interdigitated electrode are detailed in Figure S1. This laser ablation process generated LiCl-CH_3_COOK/LIG interdigitated electrodes possessing a 3D porous structure. The electrode assembly was then peeled from the glass substrate, completing the fabrication of the humidity-sensitive electrode. Conductive silver paste was applied to secure copper leads to the electrode terminals, followed by heating at 90 °C for 15 min to achieve complete curing. This procedure ultimately produced the flexible LiCl-CH_3_COOK/LIG humidity sensor.

### 2.3. Characterization and Measurement

For a detailed morphological analysis, encompassing both surface and internal features, the electrode material was subjected to investigation by Scanning Electron Microscopy (SEM, MIRA LMS, TESCAN, Brno, Czech Republic). For the elucidation of chemical states and electronic structures, analyses were performed using X-ray Photoelectron Spectroscopy (XPS, Thermo Scientific K-Alpha, Waltham, MA, USA) and confocal Raman spectroscopy (HORIBA LabRAM HR Evolution, Palaiseau, France) with a 532 nm laser.

Discrete relative humidity (RH) levels were established by inducing vapor–liquid equilibrium in stoppered flasks filled with supersaturated solutions of various salts, including LiCl, CH_3_COOK, MgCl_2_, K_2_CO_3_, NaBr, CuCl_2_, NaCl, KCl, and K_2_SO_4_. The resultant RH values of 11%, 23%, 33%, 43%, 58%, 68%, 75%, 84%, and 97% were thereby obtained (Figure S2), which were calibrated using a commercial humidity meter (CEM DT625, Dongguan, China). Before formal measurements were conducted, the accuracy of the sensors was ensured through a preconditioning procedure in which two complete cycles of exposure to the different humidity atmospheres were performed.

A controlled environment was maintained throughout all experimental operations, with temperature regulation at 25 ± 2 °C under standard atmospheric pressure, while ambient moisture levels were stabilized at 55 ± 3% RH to ensure that environmental factors would not interfere with measurements. The performance assessment of the humidity-sensing device was accomplished through sensitivity (*S*) as the principal evaluative metric, with its calculation defined by the formula *S* (%) = Δ*C*/*C*_0_ × 100%. Within this expression, the term Δ*C* is designated as the differential in capacitance values (*C*_97_–*C*_11%_), wherein the reference capacitance measured at 11% RH is represented by *C*_0_ (or *C*_11%_), and the capacitance obtained at 97% RH is indicated by *C*_97%_. Electrical property characterization of the humidity-sensing devices was performed utilizing a TH2832 LCR meter (manufactured by Tonghui, Changzhou, China) with operation parameters set at 1 V alternating current (AC). The electrochemical impedance spectroscopy of the device was carried out through the employment of a DH7003 electrochemical workstation (manufactured by Donghua, Taizhou, China).

## 3. Results and Discussion

### 3.1. Morphological and Structural Characterization

**Figure 2:**
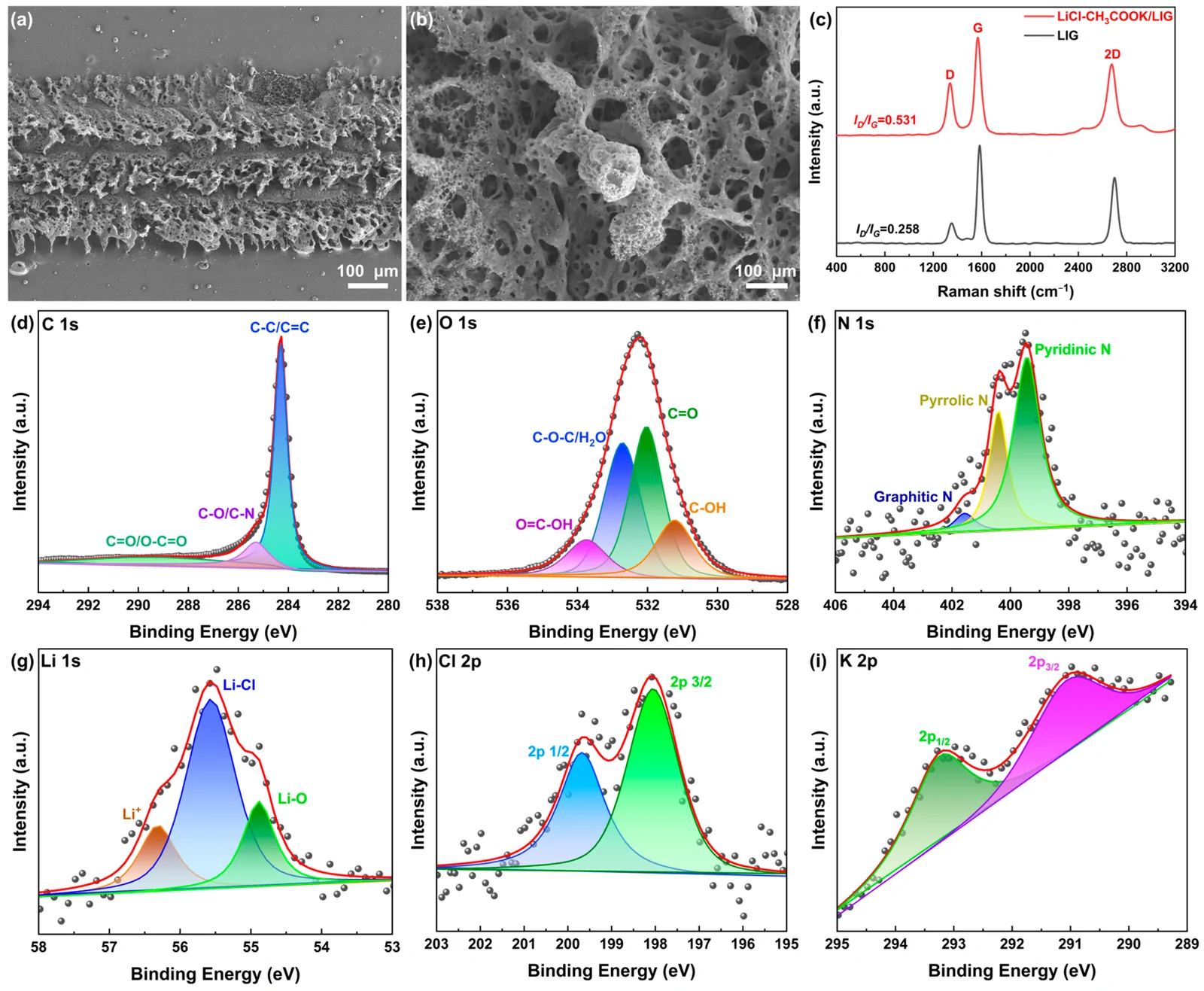
The morphological and structural characterization of electrode materials. (**a**,**b**) SEM images of LiCl-CH_3_COOK/LIG at low and high magnifications. (**c**) Raman spectra of LiCl-CH_3_COOK/LIG and pristine LIG. XPS characterization of LiCl-CH_3_COOK/LIG: (**d**–**i**) High-resolution XPS spectra of the C 1s, O 1s, N 1s, Li 1s, Cl 2p, and K 2p regions, respectively.

SEM morphological characterization reveals that the as-prepared LiCl-CH_3_COOK/LIG electrode material exhibits a continuous and well-ordered multilayer porous structure extending along the laser scribing direction at low magnification (Figure 2a). The prominent interconnected pores within the structure not only endow the material with excellent mechanical flexibility but also provide sufficient space for composite salt loading and water vapor transport. At high magnification (Figure 2b), the material further displays a 3D porous network composed of graphene sheets featuring characteristic wrinkles. This high-specific-surface-area architecture significantly increases the active interface for humidity-sensing response and offers abundant anchoring sites for LiCl and CH_3_COOK. Additionally, a small number of agglomerated salt particles are observed to adhere firmly and stably onto the graphene porous framework, confirming the favorable integration between the composite salts and the LIG substrate. This robust loading configuration effectively prevents salt loss and structurally ensures both the humidity-sensing performance and long-term reliability of the sensor.

Figure 2c presents the Raman spectra of LiCl-CH_3_COOK/LIG and pristine LIG, both exhibiting a prominent D peak (defect peak), G peak (graphitization peak), and 2D peak at approximately 1350 cm^−1^, 1580 cm^−1^, and 2700 cm^−1^, respectively, confirming that the laser induction process successfully produces few-layer graphene frameworks with favorable crystallinity [28]. The G peak originates from the in-plane stretching vibration of *sp*^2^ carbon, while the sharp single 2D peak indicates that both materials retain monolayer or few-layer graphene characteristics [28,29]. The D peak reflects lattice defects and disorder degree [30]. After doping, the intensity ratio of the D peak to the G peak (*I_D_/I_G_*) increases significantly from 0.258 to 0.531, indicating that the introduction of LiCl and CH_3_COOK nanoparticles generates more defect sites in the graphene lattice. This phenomenon likely results from lattice perturbation induced by ion intercalation, altered laser carbonization pathways, and increased edge proportions. However, the G peak position shows no apparent shift and the 2D peak maintains its single-peak morphology, confirming that doping does not disrupt the fundamental sp^2^ framework structure of graphene. The weak satellite peak near the 2D peak primarily originates from the defect-activated D + G combination mode (~2950 cm^−1^), which becomes more pronounced in doped samples with higher defect densities [28]. Additional contributions may include resonance condition variations in two-phonon processes caused by doping-induced electronic structure modulation and stretching vibrations of trace residual C-H bonds. The increase in these defect sites correlates closely with the formation of three-dimensional porous structures, providing abundant active sites and transport channels for rapid water molecule adsorption and desorption. Further observation (Figure S3) reveals that the peaks near approximately 465, 620, 1020, 1050, and 2940 cm^−1^ correspond to the characteristic Raman peaks of the CH_3_ and COO groups in potassium acetate [31].

The survey XPS spectrum of LiCl-CH_3_COOK/LIG (Figure S4) confirms the presence of C, O, N, Li, Cl and K. The high-resolution C 1s spectrum (Figure 2d) resolves a dominant *sp*^2^ C=C/C–C component at 284.2 eV, which corresponds to the graphitized core carbon network, an *sp*^3^ C–C/C–O/C–N component at 285.2 eV, which reflects lattice defects and heteroatom bonding generated by the transient extreme heating of the laser, and an O–C=O carbonyl peak at 289.5 eV, which arises from precursor decomposition or high temperature oxidation [19,32].

Deconvolution of the O 1s region (Figure 2e) further identifies four oxygen-containing functionalities. A C–OH hydroxyl component at approximately 531.2 eV forms at edge defects through carbon dangling bonds. A C=O carbonyl component at approximately 532.0 eV originates from pyrolytic carbonization. A C–O–C/H_2_O epoxy or ether component at approximately 532.7 eV incorporates a pronounced adsorbed water contribution, which is attributed to the strongly hygroscopic salts in combination with the porous architecture. An O=C–OH carboxyl component at approximately 533.8 eV distributes across deep oxidized defect sites [33,34].

Based on the N 1s spectrum (Figure 2f), the carbon matrix’s polarity and the density of active sites are seen to be cooperatively enhanced by the introduction of three distinct in situ doped nitrogen configurations. Pyridinic nitrogen at approximately 399.4 eV contributes one *p* electron to the *π* conjugated system. Pyrrolic nitrogen at approximately 400.3 eV accompanies a high degree of disorder and contributes two p electrons. Graphitic nitrogen at approximately 401.6 eV substitutes for carbon atoms within the six membered rings [35,36].

The Li 1s spectrum (Figure 2g) distinguishes three chemical environments for lithium. An ionic Li–Cl component at 55.6 eV is assigned to unreacted or recrystallized LiCl. A Li–O oxide or hydroxide component at 54.9 eV corresponds to lithium coordinated with oxygen-containing functionalities. A high binding energy component at 56.3 eV is assigned to free Li^+^ that is electrostatically adsorbed or intercalated within porous defect sites [37,38].

The Cl 2p spectrum (Figure 2h) displays a spin–orbit doublet comprising Cl 2p_3/2_ at approximately 198.0 eV and Cl 2p_1/2_ at approximately 199.6 eV with a splitting of about 1.6 eV. This signal is characteristic of inorganic chloride and confirms that chlorine persists as Cl^−^, interacts with Li^+^ and remains stable within the porous network without forming covalent C–Cl bonds [39].

The K 2p spectrum (Figure 2i) exhibits a doublet consisting of K 2p_3/2_ at approximately 291.0 eV and K 2p_1/2_ at approximately 293.8 eV with a splitting of 2.4 eV. This feature is attributed to a single K–O bonding state that arises from the interaction of K^+^ with surface oxygen-containing functionalities or in situ generated species [40,41].

### 3.2. Sensing Characteristics

The LiCl-CH_3_COOK/LIG humidity sensor primarily reflects ambient humidity levels through monitoring variations in capacitive response, and this sensing process depends strongly on the testing AC frequency (Figure S5). The intrinsic dielectric response is governed entirely by the Maxwell–Wagner–Sillars (MWS) interfacial polarization mechanism, which exhibits pronounced frequency dependence [42,43]. At low frequency (100 Hz), water molecules adsorbed in a high-humidity environment promote the dissociation of salts to generate abundant free ions. These ions undergo long-range directional migration within sufficiently long electric field cycles and accumulate substantially at heterogeneous interfaces, thereby triggering a rapidly increasing significant capacitive response. Conversely, when the alternating frequency rises to high values (10 kHz and above), the relatively large ions cannot follow the rapid reversal of the electric field polarity owing to their lower mobility. This prevents the effective establishment of interfacial polarization, whereupon the humidity-sensing response becomes markedly suppressed and the capacitance variation remains extremely weak. On the basis of the above analysis of the frequency-dependent mechanism, 100 Hz is identified as the optimal operating frequency for the device. Accordingly, all subsequent experiments employed an AC power source at a fixed frequency of 100 Hz and a voltage of 1 V.

To determine the optimal inorganic salt ratio in LiCl-CH_3_COOK/LIG composite electrodes, the mass ratio of PAA precursor to LiCl was fixed at 10:0.1 based on preliminary investigations, while the amount of CH_3_COOK was systematically varied. Composite electrodes with PAA:LiCl:CH_3_COOK mass ratios of 10:0.1:0.01, 10:0.1:0.03, 10:0.1:0.05, and 10:0.1:0.07 were fabricated via laser direct writing technology. Figure S6 presents the dynamic response and recovery characteristics of humidity sensors based on these electrodes. The testing protocol involved stepwise reduction in RH from 97% to 11%, followed by reverse elevation from 11% RH back to 97% RH. The relative capacitance change (Δ*C*/*C*_0_) of all sensors exhibits significant stepwise decrease with decreasing ambient humidity and gradual recovery during humidity elevation, demonstrating highly consistent variation trends. This indicates that LiCl-CH_3_COOK/LIG humidity sensors with different ratios display excellent reversibility and response consistency. Among these compositions, the LiCl-CH_3_COOK/LIG (10:0.1:0.03) humidity sensor exhibits optimal humidity-sensing performance, with capacitance response amplitude substantially higher than other compositions. Given its superior sensitivity, this electrode composition was selected as the optimal formulation for subsequent humidity sensing performance characterization.

To rigorously validate the synergistic deliquescence of the binary-salt system, single-salt reference devices (LiCl-only and CH_3_COOK-only) were fabricated and tested under identical conditions. As shown in Figure S7, the binary-salt (LiCl-CH_3_COOK) sensor demonstrates significantly superior humidity sensing performance compared to both single-salt devices, proving that their combination genuinely enhances moisture absorption and ion mobility beyond the capabilities of either salt alone.

**Figure 3:**
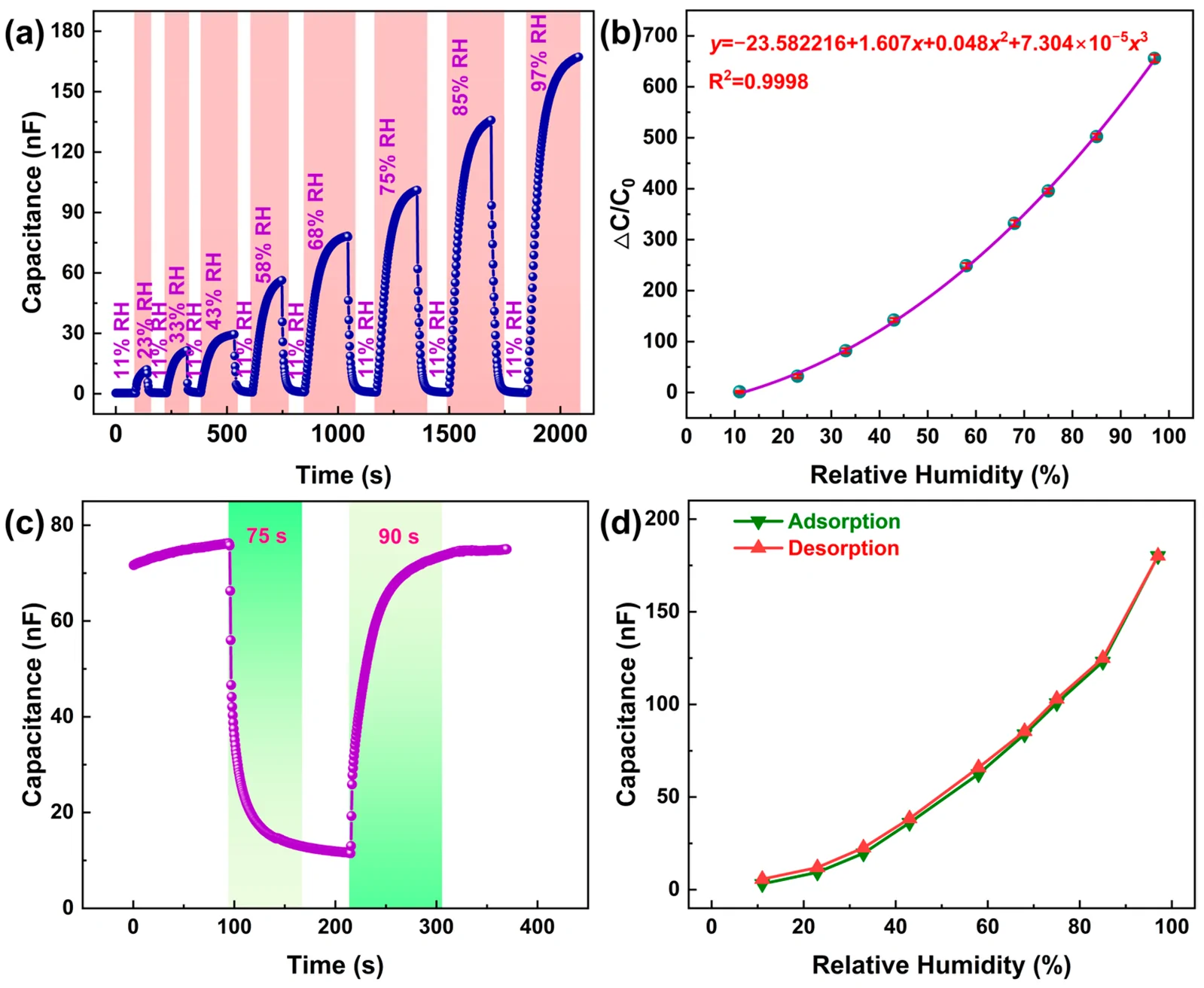
Characterization of the humidity sensing properties exhibited by the LiCl-CH_3_COOK/LIG sensor. (**a**) Dynamic capacitance response of the sensor exposed to various RH levels ranging from 11% to 97%. (**b**) The dependence of sensor capacitance on RH. (**c**) Determination of the response and recovery time durations for the sensor. (**d**) Humidity hysteresis loop observed for the sensor.

To evaluate the transient sensing performance, the dynamic response of the LiCl-CH_3_COOK/LIG humidity sensor was investigated across a wide RH range from 11% to 97% (Figure 3a). With a progressive rise in ambient humidity (from 23% to 97% RH), the sensor exhibits a distinct and stepwise escalation in its capacitance response, peaking at the highest tested humidity of 97% RH. Upon switching back to the low-humidity environment (11% RH), the capacitance swiftly and completely restores to its initial baseline value. These experimental findings demonstrate that the LiCl-CH_3_COOK/LIG humidity sensor possesses both exceptional sensitivity to rapid humidity fluctuations and a broad operational detection range.

As shown in Figure 3b for the LiCl-CH_3_COOK/LIG humidity sensor, the progression from 11% to 97% RH is marked by a dramatic rise in relative capacitance (Δ*C*/*C*_0_). To evaluate reproducibility and device-to-device variation, three independent devices (*N* = 3) were tested. Specifically, the baseline capacitance (*C*_0_) at 11% RH was measured at 0.2743 nF, with the absolute capacitance change (Δ*C*) reaching 179.8585 nF at 97% RH. This pronounced responsiveness culminates in an exceptional sensitivity of 65,570% (Δ*C*/*C*_0_). A mathematical relationship between relative humidity (*x*) and relative capacitance change (*y*) is established by the exponential function *y* = −23.582216 + 1.607*x* + 0.048*x*^2^ + 7.304 × 10^−5^*x*^3^, with the accuracy of this fit being confirmed by an excellent coefficient of determination (R^2^) of 0.9998.

As illustrated in Figure 3c, the response-recovery performance of a representative LiCl-CH_3_COOK/LIG humidity sensor is evaluated. A conventional definition is employed, wherein the time intervals for reaching 90% of the full response during the processes of moisture adsorption and desorption are designated as the response and recovery times, respectively. Following this protocol, a response duration of 75 s and a recovery period of 90 s are obtained.

Figure S8 presents the extended operational stability of the LiCl-CH_3_COOK/LIG humidity sensor over 15 consecutive days under three distinct humidity conditions: low (11% RH), medium (58% RH), and high (85% RH). The data demonstrate that the sensor exhibits excellent signal durability and clear gradient discrimination across all three humidity ranges, with signal fluctuations remaining below 3%. This performance successfully overcomes the critical bottleneck of conventional salt-based humidity-sensitive materials, which are prone to deliquescence and loss under high humidity conditions. The exceptional stability originates from the unique micro-nanoelectrode structure of the device. Through in situ laser scribing processing, LiCl-CH_3_COOK composite salt nanoparticles are uniformly encapsulated and confined within the 3D interconnected porous LIG framework. This architecture effectively suppresses the dissolution and detachment of active salts during prolonged moisture absorption. Simultaneously, the continuous and intact carbon network provides highly stable pathways for both electron and ion transport. These results strongly confirm the practical value of the device for reliable long-term operation in complex atmospheric environments.

A key benchmark for the assessment of humidity sensor reliability is hysteresis (*H*). Its quantification is achieved via the formula *H* = ±Δ*H_max_*/(2*F_RH_*), in which the maximum capacitance discrepancy between adsorption and desorption cycles is represented by Δ*H_max_*, and the full range of capacitance variation from 11% to 97% RH is denoted by *F_RH_* [44]. For a representative LiCl-CH_3_COOK/LIG humidity sensor, an approximate hysteresis value of 0.981% is ascertained from its corresponding curve (Figure 3d), a value indicative of low hysteresis characteristics.

To evaluate the selectivity of the LiCl-CH_3_COOK/LIG humidity sensor, cross-sensitivity tests were conducted against various common interfering gases, specifically NH_3_, C_2_H_5_OH, CO_2_, and O_2_. As shown in Figure S9, the sensor exhibits a significant response exclusively to water vapor, while the responses to all other tested gases remain negligible. These results demonstrate that the sensor possesses excellent selectivity for humidity detection, confirming its capability to operate reliably without interference from these common environmental gaseous species.

To evaluate the temperature dependence of the humidity sensor, Figure S10 displays the capacitance response of the LiCl-CH_3_COOK/LIG sensor at a constant 75% RH across 15–90 °C. Although the overall heating process induces a continuous capacitance decrease driven by thermally accelerated water desorption, the sensor maintains high stability within the 15–40 °C regime. Within this window, a minor capacitance drop caused by the volatilization of shallow physisorbed water is temporarily counteracted by elevated thermal energy, which significantly enhances the mobility of dissolved ions (Li^+^, K^+^, Cl^−^, and CH_3_COO^−^), thereby boosting interfacial charge accumulation to offset the signal attenuation. However, upon exceeding the thermodynamic critical point of 40.1 °C, massive water evaporation leads to severe solvent depletion. This forces the recrystallization of LiCl and CH_3_COOK, completely obstructing free ion migration and disrupting interfacial charge transport, which, coupled with the rapid loss of high-dielectric-constant water molecules, ultimately triggers a sharp plunge in capacitance.

### 3.3. Sensing Mechanism

**Figure 4:**
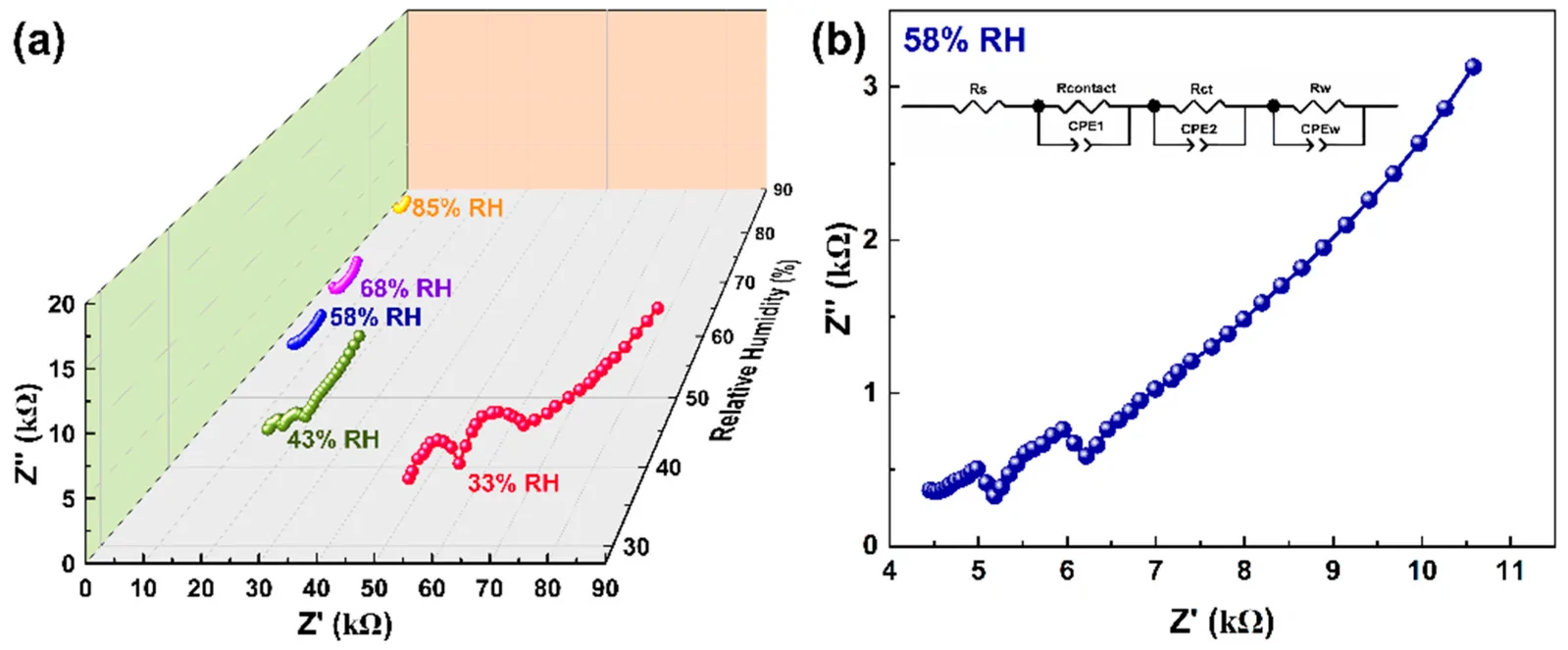
EIS analysis of the LiCl-CH_3_COOK/LIG humidity sensor. (**a**) Nyquist spectra collected under a range of RH conditions. (**b**) Nyquist plot at 58% RH and the equivalent circuit.

In conjunction with EIS data, the present study systematically elucidates the moisture sensing mechanism of the LiCl-CH_3_COOK/LIG capacitive humidity sensor. As shown in Figure 4a, across the ambient humidity range of 33% to 85% RH, the Nyquist plots of the sensor exhibit highly regular profiles (the corresponding individual detailed Nyquist plots are provided in Figure S11). With increasing humidity, the curves contract markedly and synchronously toward the origin along both the real (Z′) and imaginary (Z″) axes, and the total electrode impedance decreases rapidly from an extremely high value at 33% RH. This behavior directly reflects the superior moisture uptake and conductivity enhancement afforded by the composite electrode. To further deconvolute the kinetic processes within this system, Figure 4b presents a representative EIS measured at 58% RH and its corresponding equivalent circuit model. The model is constructed from a substrate resistance (*R_s_*), followed by two parallel circuit elements: one representing the contact/grain-boundary resistance (*R_contact_*) with a constant phase element (*CPE*_1_), and another representing the charge transfer resistance (*R_ct_*) with a corresponding constant phase element (*CPE*_2_). The final component, representing diffusion behavior, is a Warburg-related resistance (*R_w_*) associated with a constant phase element (*CPE_w_*) [45]. The distinctive double semicircle feature in the high-frequency region reveals the presence of two relaxation processes with markedly different time constants. Revised according to typical electron transport behaviors, the first semicircle at the highest frequencies is primarily attributed to the contact resistance between the LIG and current collectors, alongside grain-boundary electron transfer within the porous carbon network. In contrast, the second semicircle in the sub-high frequency region governs the charge transfer process at the interface between the LIG and the composite salt hydration film.

The distinctive double semicircle feature in the high frequency region reveals the presence of two relaxation processes with markedly different time constants. The first semicircle at the highest frequencies is attributed to the rapid bulk electron transport within the porous carbon network of the LIG, whereas the second semicircle in the sub-high frequency region governs the charge transfer process at the interface between the LIG and the composite salt hydration film. As the ambient humidity rises, LiCl and the highly deliquescent CH_3_COOK undergo a pronounced synergistic deliquescence effect, whereby water molecules are rapidly adsorbed onto the surface of the 3D porous LIG framework to form a continuous liquid water film. This process not only promotes the dissociation of abundant composite ions (Li^+^, Cl^−^, K^+^ and CH_3_COO^−^), thereby sharply increasing the concentration of free ions within the composite electrode, but also engages the Grotthuss proton hopping conduction mechanism within the hydration molecular network [5]. Together these effects substantially lower the interfacial charge transfer energy barrier and lead to a marked attenuation of *R_ct_*, manifested as the pronounced shrinkage of the sub-high frequency semicircle with rising humidity. Concurrently, within the low-frequency region of the impedance spectrum, a semi-infinite diffusion process is manifested as an inclined line, a feature that is referred to as the Warburg impedance. As the liquid film thickens and the free ion concentration increases, the spatial hindrance to ion migration decreases substantially and the diffusion rate is greatly enhanced. In summary, ambient humidity drives the synergistic deliquescence of the LiCl-CH_3_COOK composite salt. This not only establishes a low-impedance multi-ion and proton conduction network, but also promotes the rapid accumulation of abundant ions at the electrode interface under a low frequency alternating electric field, thereby inducing a strong electric double-layer polarization effect. These combined processes ultimately endow the sensor with a highly sensitive capacitive response to humidity variation.

### 3.4. Applications

**Figure 5:**
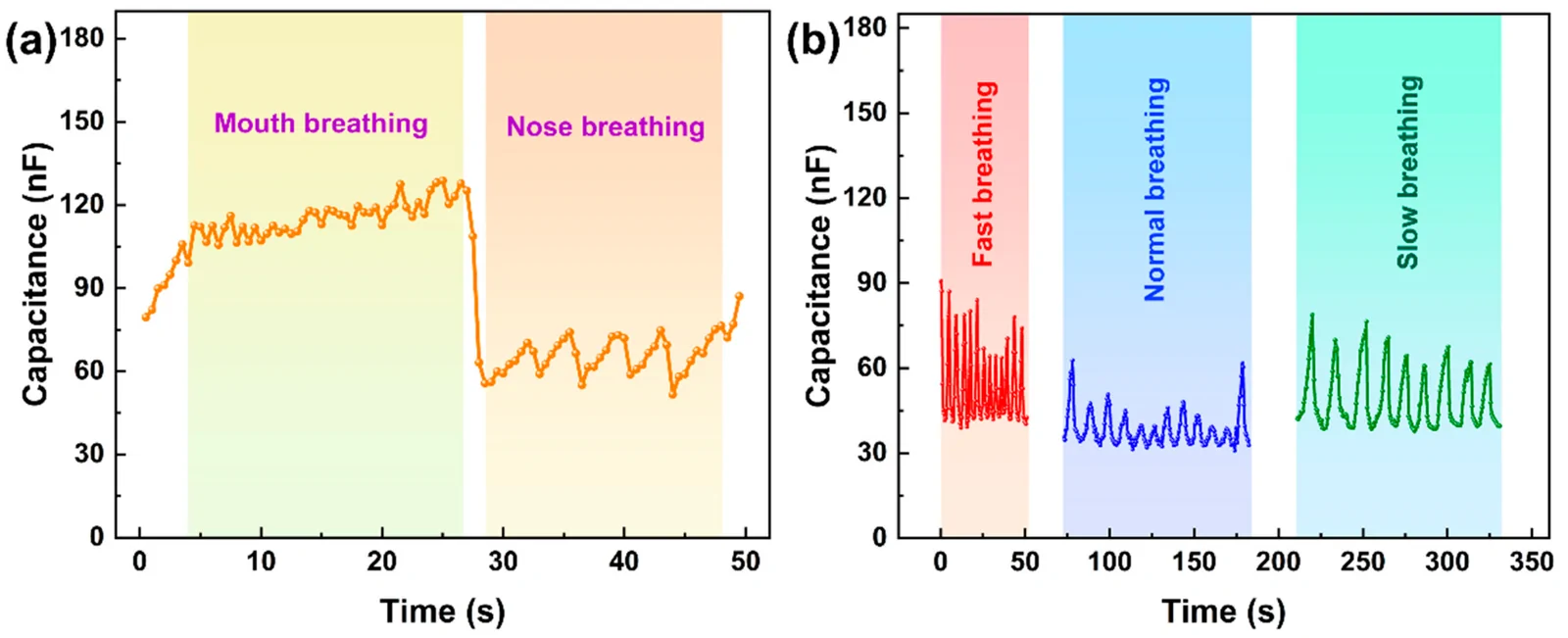
An assessment of the practical application capabilities of the LiCl-CH_3_COOK/LIG humidity sensor. (**a**) Sensor responsiveness to humidity fluctuations originating from nose and mouth breathing. (**b**) Dynamic humidity monitoring under conditions of slow, normal, and fast nasal respiration.

The practical feasibility of the LiCl-CH_3_COOK/LIG humidity sensor for real-time human respiration monitoring is demonstrated in Figure 5, where both the mode and rhythm of breathing are accurately captured. As shown in Figure 5a, a significantly higher capacitance signal intensity is generated by oral breathing compared to nasal breathing, and this pronounced discrepancy is primarily attributed to the elevated moisture content derived from oral saliva. Since the nasal route is typically used for breathing but oral breathing is adopted during intense exercise or nasal obstruction, physiological and pathological respiratory shifts can be precisely monitored through this distinct responsiveness. The dynamic tracking capabilities of the sensor under varying respiratory frequencies are illustrated in Figure 5b, where distinct, regular response peaks are successfully output for each independent inhalation and exhalation cycle across fast, normal, and slow breathing patterns. Real-time breathing rate is reflected by this consistent peak formation, highlighting the exceptional sensitivity and fast transient responsiveness of the device. Notably, while the sensor requires 75/90 s to reach 90% of its stable signal change during a large-signal humidity transition, signal overlapping is effectively prevented even during rapid respiration. During such continuous respiration, the breathing cycle is too short for the sensor to reach full macroscopic adsorption/desorption equilibrium. Instead, the sensor effectively captures the small-signal, transient humidity fluctuations caused by exhalation. Its highly sensitive initial slope allows it to distinctly resolve individual breathing cycles only seconds apart, despite the longer equilibrium time. These results validate the sensor’s reliability in distinguishing complex respiratory behaviors, highlighting its tremendous potential for integration into next-generation wearable personal health monitors and clinical respiratory diagnostic systems.

**Figure 6:**
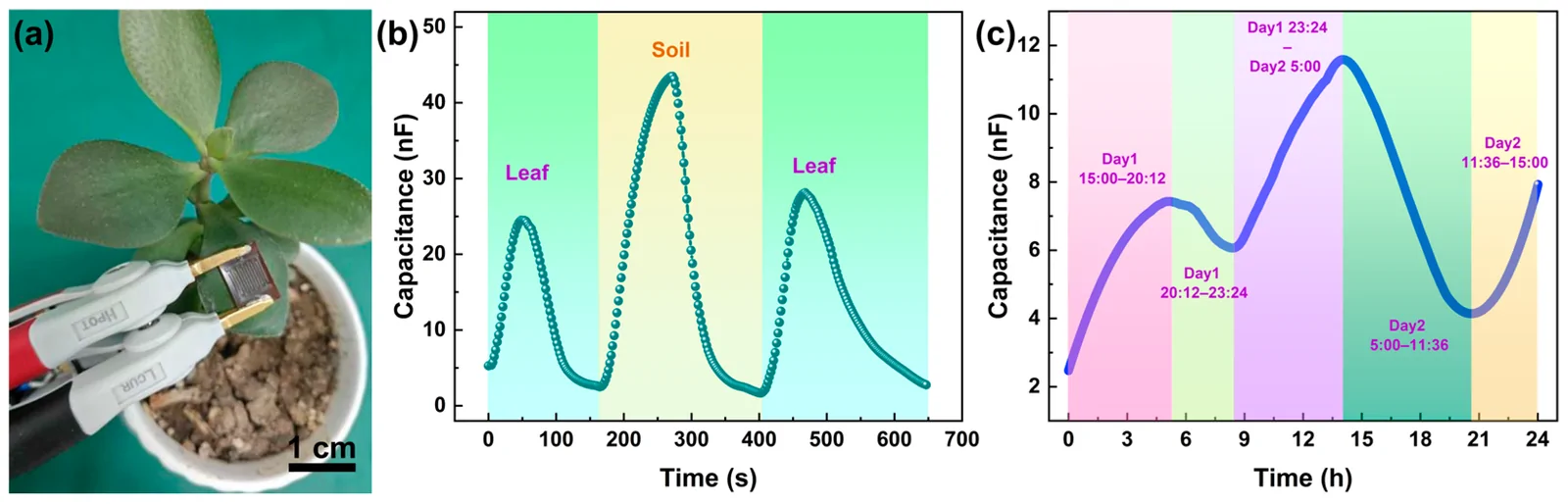
Application of the LiCl-CH_3_COOK/LIG flexible humidity sensor in smart agriculture monitoring. (**a**) Photograph of the flexible sensor attached to a potted plant. (**b**) Real-time capacitance response alternating between leaf and soil. (**c**) Continuous 24 h monitoring of leaf humidity across day-night cycles.

To verify the application potential of the LiCl-CH_3_COOK/LIG flexible humidity sensor in smart agriculture, potted green plants served as the test subjects, enabling systematic evaluation of the sensor’s capacity to monitor humidity across different plant growth regions and under long-term natural diurnal cycling conditions (Figure 6a). Under a room-temperature constant environment, the sensor was alternately attached to leaf and soil surfaces, and its capacitive response characteristics were continuously measured to examine short-term spatial humidity resolution (Figure 6b). The leaf surface exhibited capacitance within a lower range owing to the influence of water vapor evaporation, whereas the soil environment presented higher ambient humidity and abundant moisture. Following sufficient moisture absorption by the humidity-sensitive layer, the capacitance increased markedly, with smooth peak-to-valley transitions and no signal noise, thereby precisely distinguishing humidity differences among distinct regions.

To further assess the practicality and reliability of the LiCl-CH_3_COOK/LIG sensor under long-term complex conditions, 24 h continuous in situ humidity monitoring was conducted on green plant leaves. This test fully encompassed diurnal cycling and variations in plant physiological rhythms, and the capacitive response curve (Figure 6c) displays periodic fluctuations that correspond closely with plant physiological activity. During daytime, sufficient illumination enhanced transpiration and the capacitance rose steadily. At night, the temperature decreased and transpiration nearly ceased, while moisture retained on the leaf surface condensed into water vapor, causing the capacitance to reach its daily peak. By the following early morning, as the temperature rose and photosynthesis resumed, the capacitance declined once more. The variations across each period corresponded closely with the actual humidity patterns of the leaf surface, and the pattern observed during the second daytime remained consistent with that of the first day, demonstrating favorable repeatability. Throughout the entire monitoring period, the signal remained stable and the data distribution appeared regular, enabling precise capture of subtle changes in leaf surface humidity. These results indicate that the sensor possesses excellent multi-region synchronous monitoring capability and long-term operational stability, and that it faithfully reflects humidity variation patterns across different plant regions and over full-day timescales. The sensor effectively overcomes the limitations of conventional sensors, including susceptibility to detachment, signal instability and long-term data distortion, thereby providing reliable technical support for dynamic assessment of plant growth status, precision irrigation control and crop growth monitoring in smart agriculture.

## 4. Conclusions

In summary, this study successfully demonstrates an in situ laser scribing strategy to prepare LiCl-CH_3_COOK/LIG composite electrodes for high-performance flexible capacitive humidity sensors. By harnessing the synergistic combination of a 3D porous LIG conductive scaffold and spatially confined hygroscopic salts, the resulting device overcomes the long-standing bottlenecks of salt leaching and electrode delamination that plague conventional inorganic salt-based sensors. The optimized composite electrode delivers a substantial humidity sensitivity of 65,570% (Δ*C*/*C*_0_), together with rapid kinetics and negligible hysteresis, which outperforms most reported flexible humidity sensors. The underlying sensing mechanism is attributed to the establishment of a low-impedance multi-ion and proton conduction network via synergistic deliquescence, coupled with pronounced interfacial polarization under low-frequency electric fields. The streamlined, mask-free fabrication process further ensures cost-effective scalability and design flexibility. Successful deployment in real-time human respiration detection and long-term plant microenvironment monitoring underscores the sensor’s practical viability in wearable healthcare and smart agriculture. This work not only advances the rational design of robust flexible sensing interfaces but also paves the way for next-generation miniaturized and highly integrated environmental monitoring platforms.

## Data Availability

The original contributions presented in this study are included in the article. Further inquiries can be directed to the corresponding author.

## Supplementary Materials

The following supporting information can be downloaded at https://www.mdpi.com/article/10.3390/nano16160996/s1. Figure S1: The geometric configuration and detailed dimensions of the interdigitated electrode. Figure S2: Saturated salt solutions as the basis for constant RH control in humidity assessments. Figure S3: Detailed Raman spectrum of LiCl-CH_3_COOK/LIG. Figure S4: XPS survey spectrum of LiCl-CH_3_COOK/LIG. Figure S5: Capacitive responses of the LiCl-CH_3_COOK/LIG humidity sensor under different frequencies. Figure S6: The influence of compositional variation on the response and recovery characteristics of the LiCl-CH_3_COOK/LIG humidity sensor. Figure S7: Comparison of humidity responses among the LiCl-CH_3_COOK/LIG sensor, LiCl-only sensor, and CH_3_COOK-only sensor. Figure S8: Long-term stability characterization of LiCl-CH_3_COOK/LIG humidity sensors under 11%, 58%, and 85% RH environments (representing low, medium, and high humidity). Figure S9: The selectivity of the LiCl-CH_3_COOK/LIG humidity sensor. Figure S10: Temperature-dependent capacitance characteristic of the LiCl-CH_3_COOK/LIG humidity sensor measured at 75% RH across a temperature range of 15–90 °C. Figure S11: Detailed Nyquist plots obtained at different RH levels of 33%, 43%, 58%, 68%, and 85%.

## References

[1] Lv W., Yang J., Xu Q., Mehrez J.A.-A., Shi J., Quan W., Luo H., Zeng M., Hu N., Wang T. (2024). Wide-range and high-accuracy wireless sensor with self-humidity compensation for real-time ammonia monitoring. Nat. Commun..

[2] Song S., Zhang M., Gong X., Shi S., Fang J., Wang X. (2026). Advances in Wearable Sensors for Health Management: From Advanced Materials to Intelligent Systems. Adv. Funct. Mater..

[3] Wang J., Sharma V., Nikolaeva V.O., Alimi L.O., Chen Z., Lin W.B., Khashab N.M. (2026). Smart supramolecular humidity sensors based on crystalline organic cages for noninvasive freshness and respiratory monitoring. Adv. Mater..

[4] Xiao C., Liu X., Zhao Y., Huang C., Zhou N., Mao H. (2025). Flexible humidity sensors for diverse applications. Microsyst. Nanoeng..

[5] Lu Y., Yang G., Shen Y., Yang H., Xu K. (2022). Multifunctional Flexible Humidity Sensor Systems Towards Noncontact Wearable Electronics. Nano-Micro Lett..

[6] Lao S., Duan Z., Zhao Q., Yuan Z., Jiang Y., Tai H. (2025). Recent advances in mass-sensing humidity sensors: Mechanisms, materials, and applications. Nanoscale.

[7] Mahaseth D.N., Islam T. (2024). An ultrafast capacitive humidity sensor with excellent static and dynamic characteristics. IEEE Sens. J..

[8] Fujii T., Takita M., Suzuki T., Nomura M. (2026). Development of a Capacitive Humidity Sensor Based on Polyimide/Carbon Nanotube (CNT) Composite Thin Films with High CNT Content. Sens. Mater..

[9] Ren J., Dan R., Guo Y., Zhao J. (2026). A Flexible Capacitive Humidity Sensor Enabled by LIG-Anchored Synergistic GO-PEDOT:PSS-MXene Composite. Materials.

[10] Huang C., Chen J., Zhang Y., Jiang C., Wang Y., Qi J., Yang H., Gao K., Jiang M., Liu F. (2025). Wide-detection-range, highly-sensitive, environmental-friendly and flexible cellulose-based capacitive humidity sensor. Carbohydr. Polym..

[11] Srilarueang S., Sreejivungsa K., Thanamoon N., Jarernboon W., Thongbai P. (2024). Optimizing sintering conditions and microstructure for enhanced dielectric and humidity sensing properties of Na1/3Ca1/3Tb1/3Cu3Ti4O12 ceramics. Mater. Chem. Phys..

[12] Anisimov Y., Evitts R., Cree D., Wilson L. (2021). Polyaniline/Biopolymer Composite Systems for Humidity Sensor Applications: A Review. Polymers.

[13] Alrammouz R., Podlecki J., Vena A., Garcia R., Abboud P., Habchi R., Sorli B. (2019). Highly porous and flexible capacitive humidity sensor based on self-assembled graphene oxide sheets on a paper substrate. Sens. Actuators B.

[14] Kang J., Gao F., Wang Y., Fu J., Song S., Jin F., Bai G., Shen C. (2025). High sensitivity and fast response wireless humidity sensor enabled by MXene-Lys for finger proximity detection and health monitoring applications. Chem. Eng. J..

[15] Leng X., Wang C., Duan Z., Wang Y., Wang Q., Yuan Z., Jiang Y., Tai H. (2026). Electrochemical humidity sensors based on LiCl/carbon ink with adjustable humidity sensing and enhanced power generation performances. Sens. Actuators B.

[16] Li L., Ren J., Zhang C., Li F., Chen Z., Xu R., Zhao J. (2026). Screen-printed paper-based humidity sensors employing laser-induced graphene and potassium acetate. Sens. Actuators B.

[17] Kim J.-H., Moon B.-M., Hong S.-M. (2012). Capacitive humidity sensors based on a newly designed interdigitated electrode structure. Microsyst. Technol..

[18] Wang H., Liu X., Yu Y., Zhao J., Gao Y., Fei T., Chen Z. (2025). High-Performance Humidity Sensors Based on Hexagonal Boron Nitride Films Prepared by Magnetron Sputtering. IEEE Sens. J..

[19] Lin J., Peng Z., Liu Y., Ruiz-Zepeda F., Ye R., Samuel E.L.G., Yacaman M.J., Yakobson B.I., Tour J.M. (2014). Laser-induced porous graphene films from commercial polymers. Nat. Commun..

[20] Huang L., Su J., Song Y., Ye R. (2020). Laser-induced graphene: En route to smart sensing. Nano-Micro Lett..

[21] Lan L., Le X., Dong H., Xie J., Ying Y., Ping J. (2020). One-step and large-scale fabrication of flexible and wearable humidity sensor based on laser-induced graphene for real-time tracking of plant transpiration at bio-interface. Biosens. Bioelectron..

[22] Liu S., Chen R., Chen R., Jiang C., Zhang C., Chen D., Zhou W., Chen S., Luo T. (2023). Facile and cost-effective fabrication of highly sensitive, fast-response flexible humidity sensors enabled by laser-induced graphene. ACS Appl. Mater. Interfaces.

[23] Ye R., James D.K., Tour J.M. (2019). Laser-induced graphene: From discovery to translation. Adv. Mater..

[24] Song Y., Dan R., Li L., Xia X., Zhao J., Xu R. (2025). A flexible humidity sensor based on GO/PEDOT: PSS modified laser-induced graphene electrode. Sens. Actuators A.

[25] He B., Xia W., Deng Y., Wu J., Guo Z., Wang Z., Zhang B., Feng Y. (2025). High-Performance wearable flexible humidity sensor based on plasma-treated graphene oxide for medical monitoring and non-contact sensing. Appl. Surf. Sci..

[26] Huang Y., Hao C., Wang X., Bao Z., Liu T., Li J., Li S., Pi M., Zhang D. (2025). Capacitive humidity sensor with enhanced sensitivity based on graphene oxide/lignosulfonate with laser-induced graphene electrodes for noncontact detection. IEEE Sens. J..

[27] Sun W., Li W., Xue C., Zhang W., Jiang Y. (2025). MXene-silk composites integrated on laser-induced graphene platform for touch-free sensing. Chem. Eng. J..

[28] Paillet M., Parret R., Sauvajol J.L., Colomban P. (2018). Graphene and related 2D materials: An overview of the Raman studies. J. Raman Spectrosc..

[29] Yin X.-T., You E.-M., Zhou R.-Y., Zhu L.-H., Wang W.-W., Li K.-X., Wu D.-Y., Gu Y., Li J.-F., Mao B.-W. (2024). Unraveling the energy storage mechanism in graphene-based nonaqueous electrochemical capacitors by gap-enhanced Raman spectroscopy. Nat. Commun..

[30] Eckmann A., Felten A., Mishchenko A., Britnell L., Krupke R., Novoselov K.S., Casiraghi C. (2012). Probing the nature of defects in graphene by Raman spectroscopy. Nano Lett..

[31] Gasgnier M., Petit A. (1994). Effects of microwave and heating treatments on the crystallographic properties of a potassium acetate powder. J. Mater. Sci..

[32] Yang D., Velamakanni A., Bozoklu G., Park S., Stoller M., Piner R.D., Stankovich S., Jung I., Field D.A., Ventrice C.A. (2009). Chemical analysis of graphene oxide films after heat and chemical treatments by X-ray photoelectron and Micro-Raman spectroscopy. Carbon.

[33] Zhang J., Ren M., Wang L., Li Y., Yakobson B.I., Tour J.M. (2018). Oxidized laser-induced graphene for efficient oxygen electrocatalysis. Adv. Mater..

[34] Pei S., Cheng H.-M. (2012). The reduction of graphene oxide. Carbon.

[35] Rabchinskii M.K., Saveliev S.D., Stolyarova D.Y., Brzhezinskaya M., Kirilenko D.A., Baidakova M.V., Ryzhkov S.A., Shnitov V.V., Sysoev V.V., Brunkov P.N. (2021). Modulating nitrogen species via N-doping and post annealing of graphene derivatives: XPS and XAS examination. Carbon.

[36] Pels J., Kapteijn F., Moulijn J., Zhu Q., Thomas K. (1995). Evolution of nitrogen functionalities in carbonaceous materials during pyrolysis. Carbon.

[37] Li Y., Zhao Y., Liu X., Qiu Y., He Z., Zhang S., Fang H., Chen L., Zhang L., Shi G. (2023). 2D metallic abnormal Li2Cl crystals with unique electronic characteristics applied in capacitor and humidity sensor. Adv. Mater. Interfaces.

[38] Aurbach D., Markovsky B., Shechter A., Ein-Eli Y., Cohen H. (1996). A comparative study of synthetic graphite and Li electrodes in electrolyte solutions based on ethylene carbonate-dimethyl carbonate mixtures. J. Electrochem. Soc..

[39] Ganbold E., Kim E.S., Li Y., Yin F., Sharma P.K., Jeon J.-B., Oh J.-M., Lee D.N., Kim N.Y. (2023). Highly sensitive interdigitated capacitive humidity sensors based on sponge-like nanoporous PVDF/LiCl composite for real-time monitoring. ACS Appl. Mater. Interfaces.

[40] Kloprogge J.T., Ponce C.P., Ortillo D.O. (2021). X-ray Photoelectron Spectroscopic Study of Some Organic and Inorganic Modified Clay Minerals. Materials.

[41] Caracciolo L., Madec L., Martinez H. (2021). XPS analysis of K-based reference compounds to allow reliable studies of solid electrolyte interphase in K-ion batteries. ACS Appl. Energy Mater..

[42] Xia X., Zhong Z., Weng G.J. (2017). Maxwell–Wagner–Sillars mechanism in the frequency dependence of electrical conductivity and dielectric permittivity of graphene-polymer nanocomposites. Mech. Mater..

[43] Samet M., Kallel A., Serghei A. (2019). Polymer bilayers with enhanced dielectric permittivity and low dielectric losses by Maxwell–Wagner–Sillars interfacial polarization: Characteristic frequencies and scaling laws. J. Appl. Polym. Sci..

[44] Wang Y., Zhang L., Zhou J., Lu A. (2020). Flexible and Transparent Cellulose-Based Ionic Film as a Humidity Sensor. ACS Appl. Mater. Interfaces.

[45] Das S., Banerjee A., Nandi U., Ghosh A. (2025). Critical review on the analysis of electrochemical impedance spectroscopy data. J. Appl. Phys..

