# Supplementary material for: In Situ Inorganic Salt-Enabled Laser-Induced Graphene for High-Performance Flexible Capacitive Humidity Sensing
Source: Nanomaterials (Basel). 2026 Aug 13;16(16):996. doi: 10.3390/nano16160996 (PMC13516668; doi:10.3390/nano16160996)
Supplement: Supplementary file 1 [file nanomaterials-16-00996-s001.zip › nanomaterials-4458469-supplementary.pdf]

Supporting Information

In-Situ Inorganic Salt-Enabled Laser-Induced Graphene for High-Performance Flexible Capacitive Humidity Sensing

Jitong Ren, Zihan Li, Lei Gu, Weilu Chen, Xinyi Zhou, Yanyan Guo, Jiang Zhao**

College of Integrated Circuit Science and Engineering, Nanjing University of Posts and Telecommunications, 9 Wenyuan Road, Nanjing 210023, P. R. China

*Corresponding authors, E-mail: guoyy@njupt.edu.cn (Y. Guo); jzhao@njupt.edu.cn (J. Zhao).

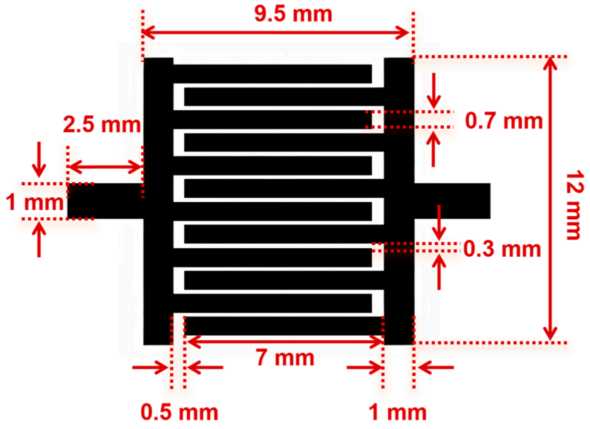

Figure S1. The geometric configuration and detailed dimensions of the interdigitated electrode.

The specific parameters of the interdigitated electrode are detailed in Figure S1.

Finger length: 7 mm;

Finger width: 0.7 mm;

Inter-electrode gap: 0.3 mm;

Number of conductive fingers: 12 fingers (6 pairs);

Active sensing area: 90 mm^2 (calculated based on the inner interdigitated region measuring 7.5 mm in width × 12 mm in height);

Total footprint area: 114 mm^2 (overall dimensions of 9.5 mm × 12 mm, including the 1 mm wide busbars).

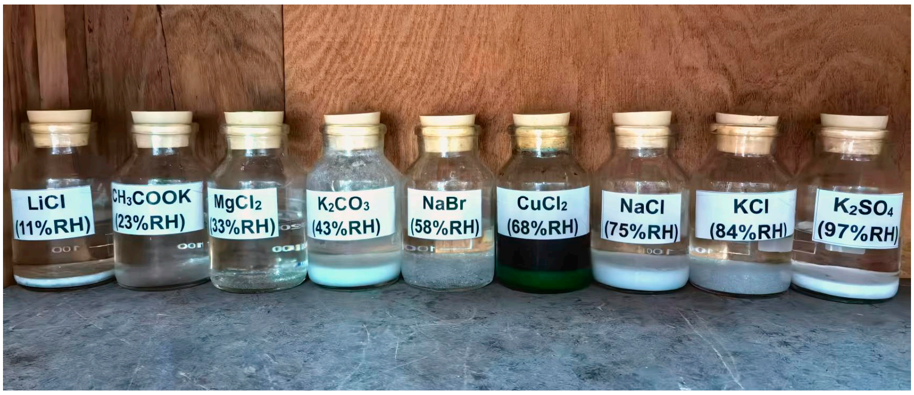

Figure S2. Saturated salt solutions as the basis for constant RH control in humidity assessments.

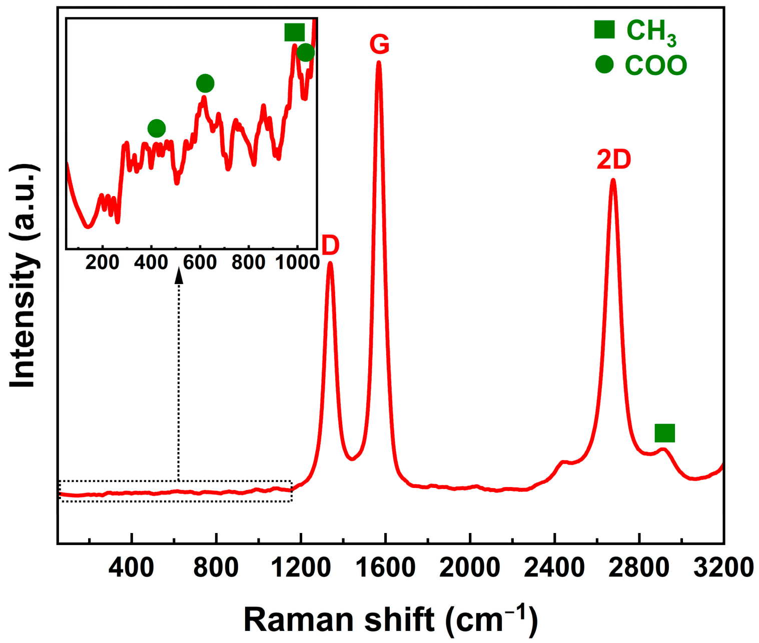

Figure S3. Detailed Raman spectrum of LiCl-CH_3COOK/LIG.

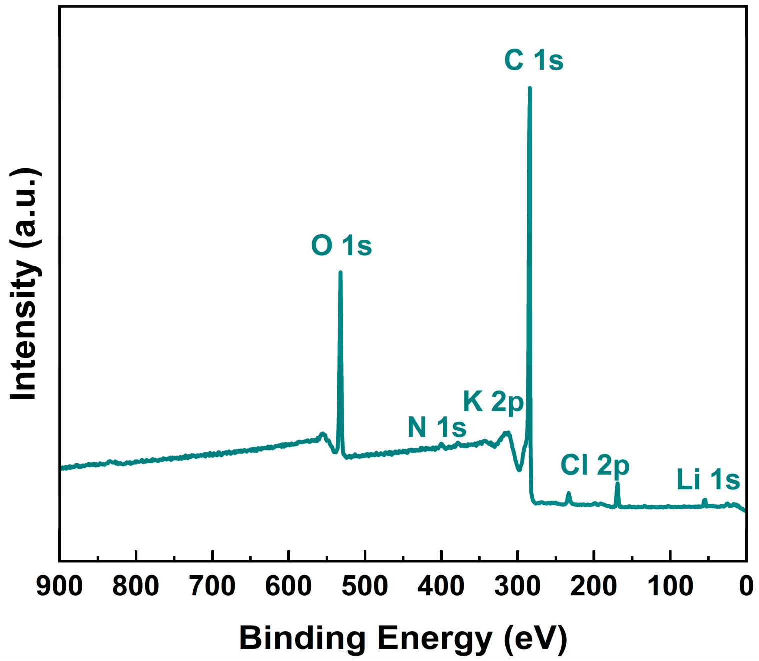

Figure S4. XPS survey spectrum of LiCl-CH_3COOK/LIG.

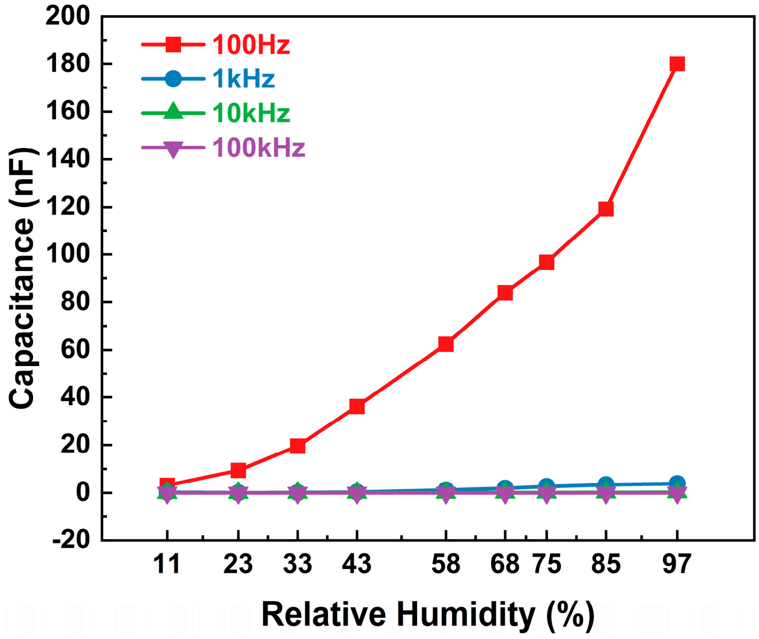

Figure S5. Capacitive responses of the LiCl-CH_3COOK/LIG humidity sensor under different frequencies.

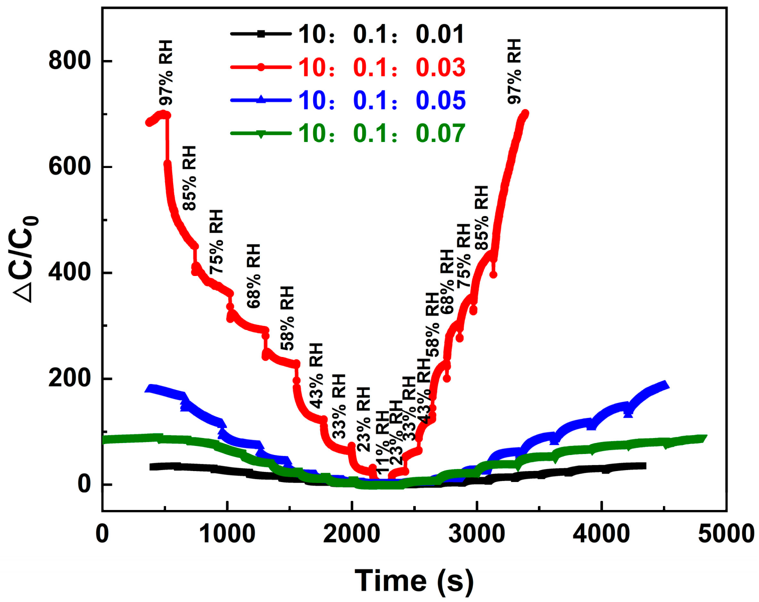

Figure S6. The influence of compositional variation on the response and recovery characteristics of the LiCl-CH_3COOK/LIG humidity sensor.

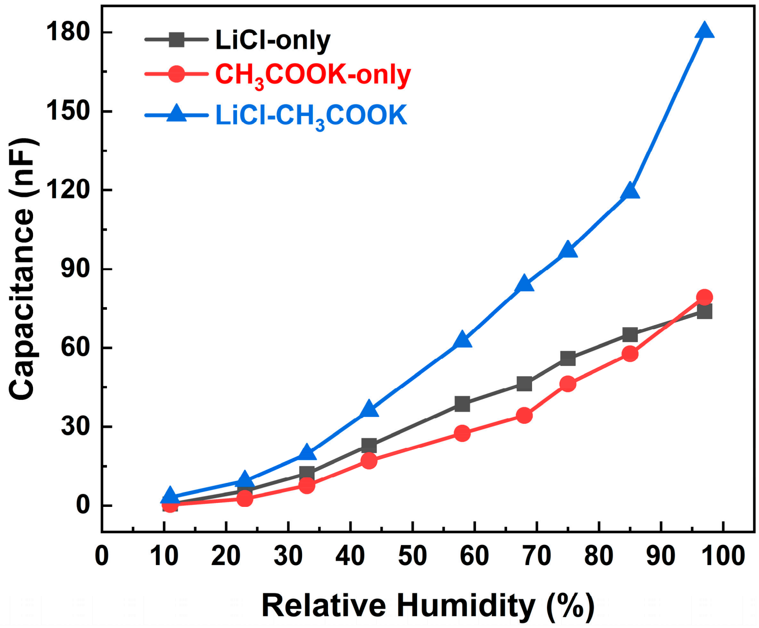

Figure S7. Comparison of humidity responses among the LiCl-CH_3COOK/LIG sensor, LiCl-only sensor, and CH_3COOK-only sensor.

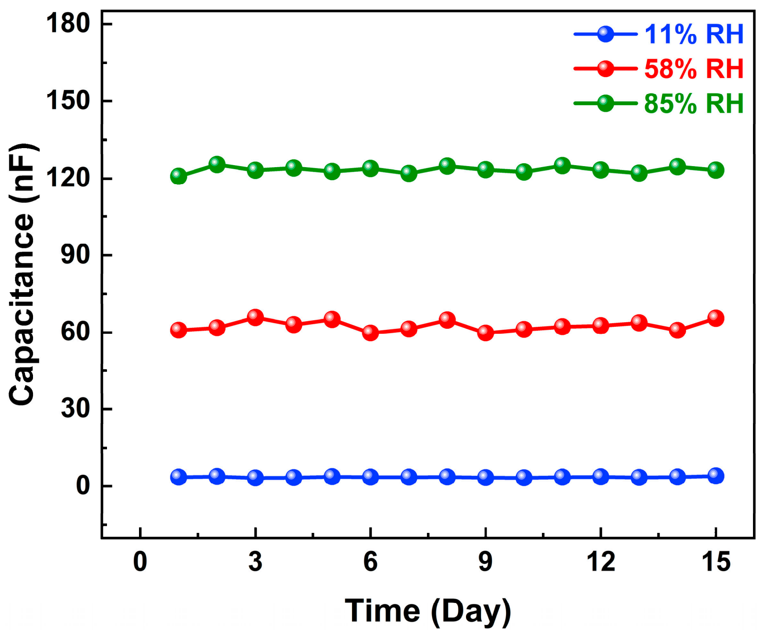

Figure S8. Long-term stability characterization of LiCl-CH_3COOK/LIG humidity sensors under 11%, 58%, and 85% RH environments (representing low, medium, and high humidity).

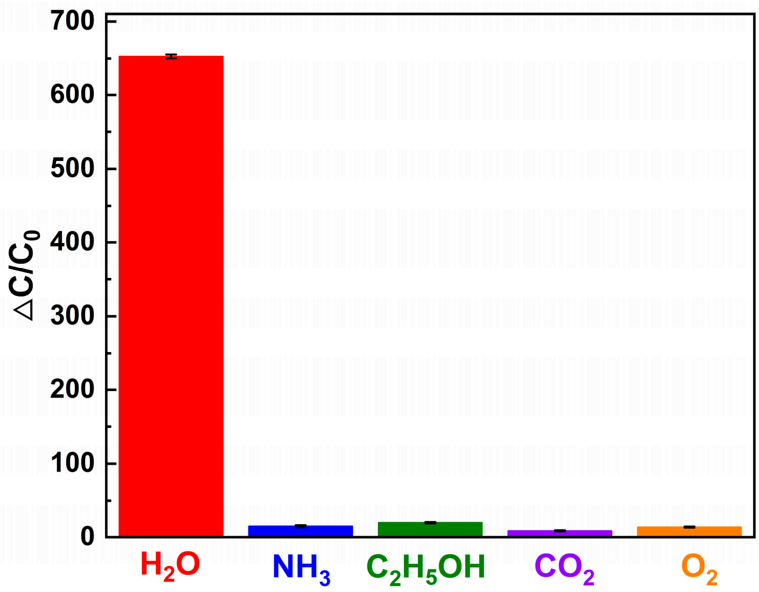

Figure S9. The selectivity of the LiCl-CH_3COOK/LIG humidity sensor.

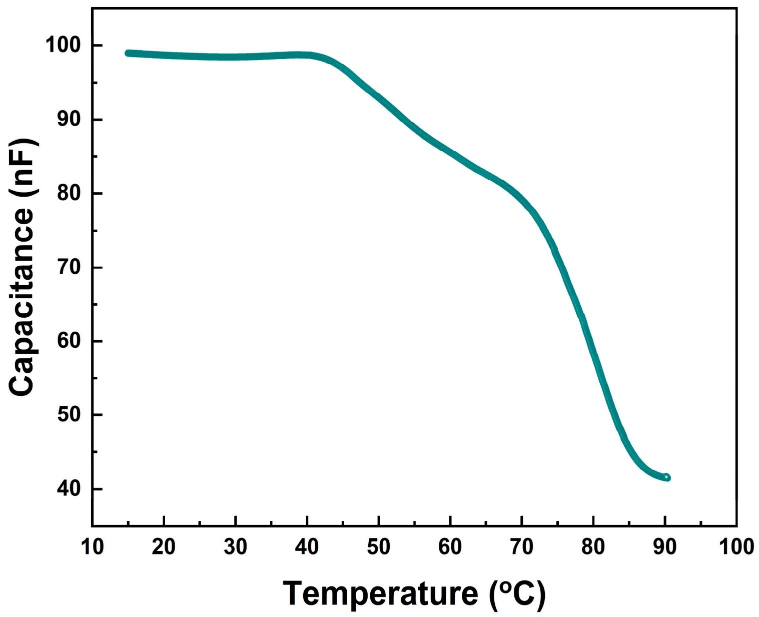

Figure S10. Temperature-dependent capacitance characteristic of the LiCl-CH_3COOK/LIG humidity sensor measured at 75% RH across a temperature range of 15–90 °C.

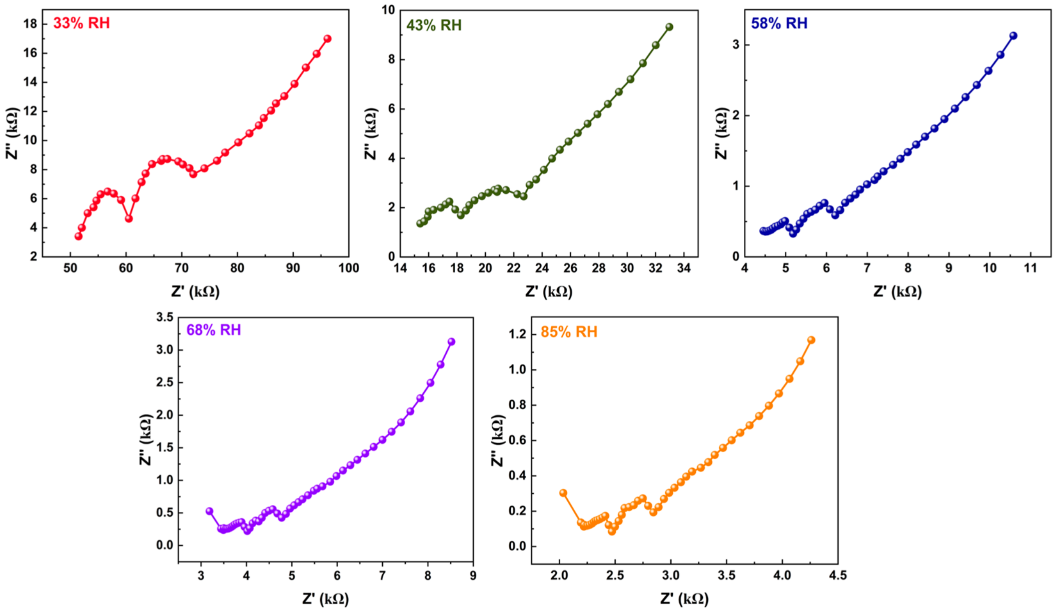

Figure S11. Detailed Nyquist plots obtained at different RH levels of 33%, 43%, 58%, 68%, and 85%.
